# The Experiences of Patients With Adjuvant and Metastatic Melanoma Using Disease-Specific Social Media Communities in the Advent of Novel Therapies (Excite Project): Social Media Listening Study

**DOI:** 10.2196/34073

**Published:** 2022-05-13

**Authors:** Guy Faust, Alison Booth, Evie Merinopoulou, Sonia Halhol, Heena Tosar, Amir Nawaz, Magdalena Szlachetka, Gavin Chiu

**Affiliations:** 1 University Hospitals of Leicester National Health Service Trust Leicester United Kingdom; 2 Evidera London United Kingdom; 3 Novartis London United Kingdom

**Keywords:** health-related social media, patient-centric, melanoma, adjuvant, metastatic, immunotherapy, targeted therapy, natural language processing, patient experience, cancer, cancer therapy, patient perspective, social media, patient experience, caregiver experience

## Abstract

**Background:**

Immunotherapy and targeted therapy treatments are novel treatments available for patients with metastatic and adjuvant melanoma. As recently approved treatments, information surrounding the patients’ and caregivers’ experience with these therapies, perceptions of treatments, and the effect the treatments have on their day-to-day life are lacking. Such insights would be valuable for any future decision-making with regard to treatment options.

**Objective:**

This study aims to use health-related social media data to understand the experience of patients with adjuvant and metastatic melanoma who are receiving either immunotherapy or targeted therapies. This study also included caregivers’ perspectives.

**Methods:**

Publicly available social media forum posts by patients with self-reported adjuvant or metastatic melanoma (and their caregivers) between January 2014 to October 2019 were programmatically extracted, deidentified, cleaned, and analyzed using a combination of natural language processing and qualitative data analyses. This study identified spontaneously reported symptoms and their impacts, symptom duration, and the impact of treatment for both treatment groups.

**Results:**

Overall, 1037 users (9023 posts) and 114 users (442 posts) were included in the metastatic group and adjuvant group, respectively. The most identified symptoms in both groups were fatigue, pain, or exanthema (identified in 5%-43% of patients dependent on the treatment group). Symptom impacts reported by both groups were physical impacts, impacts on family, and impacts on work. Positive treatment impacts were reported in both groups and covered the areas of work, social and family life, and general health and quality of life.

**Conclusions:**

This study explored health-related social media to better understand the experience and perspectives of patients with melanoma receiving immunotherapy or targeted therapy treatments as well as the experience of their caregivers. This exploratory work uncovered the most discussed concerns among patients and caregivers on the forums including symptoms and their impacts, thus contributing to a deeper understanding of the patient/caregiver experience.

## Introduction

### Background

Melanoma is a skin cancer that arises from uncontrolled proliferation of melanocytes. It is the fifth most common cancer in the United Kingdom, accounting for nearly 5% of all new cancer cases [1]. In the last 10 years, the incidence of melanoma has increased by more than 50% in the United Kingdom and is further projected to increase by 7% between 2014 and 2035 [2,3]. The worldwide incidence of melanoma has also steadily increased over the last decades, ranging between 4% and 6% in North America, Australia, and New Zealand [4].

Survival rates for melanoma depend on the disease stage; for example, 1-year net survival at stage I is similar to that of the general population; however, survival at stage IV is historically much lower [5], with the median reported at just 6 to 10 months [6]. Surgery, while effective for early stages of melanoma, is a less effective treatment option for patients with metastatic or late-stage disease [7]. Newer therapies such as immunotherapy treatments and targeted therapies (TTs) have shown good efficacy in the treatment of metastatic melanoma and have shifted the treatment paradigm [8,9]. TTs block the growth and spread of cancer by interfering with specific molecules that are involved in the growth, spread, and progression of cancer. These, however, are limited to patients who carry the BRAF V600E/K mutations, the prevalence of which in melanoma is estimated to be ~40% to 50% [10-12]. Dabrafenib plus trametinib combination therapy is routinely used as a TT and was licensed for use in metastatic melanoma with BRAF V600E or V600K mutations in August 2015 [13]. Dabrafenib with trametinib has also been recommended for adjuvant treatment of adults with resected stage III BRAF V600 mutation-positive melanoma [14]. The European Society for Medical Oncology 2019 guidelines for metastatic melanoma suggest that patients be treated with nivolumab, nivolumab/ipilimumab, or pembrolizumab in the first-line setting, and for patients with BRAF V600 mutation, vemurafenib/cobimetinib (not recommended by the National Institute for Health and Care Excellence in the United Kingdom). Dabrafenib/trametinib and encorafenib/binimetinib can also be considered [15].

While trial data on these therapies are shown to have survival benefit, there are few reports regarding patients’ experiences while undergoing treatment. Social media provides an opportunity to unveil a more personal and firsthand view on patients’ and caregivers’ perspectives and experiences with melanoma receiving treatment.

Health-related social media has substantial potential as a sizeable real-world data source due to available posts from thousands of patients and caregivers that would be hard to capture in traditional data sources. These experiences are reported in a setting with no researcher or medical professional present. Furthermore, in June 2018 the US Food and Drug Administration (FDA) encouraged the use of social media to understand the patient perspective [16]. Studies have also suggested that real-world data from social media can provide a better understanding of the patient’s behavior, quality of life, adverse events, and any episodes [17,18].

### Objectives

The objective of this study was to use publicly available health-related social media data (ie, discussions on melanoma-specific patient online forums) to understand the experience of patients with adjuvant and metastatic melanoma receiving immunotherapy or TTs and their caregivers. The reported symptoms and their associated burden such as physical impacts, impacts on family, and impacts on quality of life were of specific interest in this study.

## Methods

This was a retrospective analysis of existing publicly available discussions posted from January 2014 to October 2019 (study period) in social media forums for patients with self-reported adjuvant or metastatic melanoma and their caregivers.

### Data Source

To determine the feasibility of addressing the study objectives and to select the forums for inclusion in the study, a feasibility evaluation was conducted via a manual search and inspection of existing social media forums (finalized in May 2019). The search strategy focused on identifying melanoma-specific patient forums using relevant search terms such as “melanoma patient forums” and “melanoma discussion boards.” Generic social media forums (eg, Facebook and Twitter) were not considered due to the high level of noise (ie, irrelevant material).

Searches for social media forums were conducted using the Google Search engine for both the United Kingdom and the United States to get a complete picture of the available melanoma forum landscape. The first five pages of results were screened by title, and relevant forums were summarized.

Disease-specific social media forums were selected based on their relevance to disease experience, user profile (melanoma patients or caregivers), being currently active (ie, multiple posts in recent months to accurately reflect the most up-to-date discussions among parents/caregivers), posts in the English language, and material being freely available for anyone to access and read, with no registration required. No geographical restrictions were applied when selecting the social media forums.

Based on these criteria, forums from the following social media forums were included: Melanoma International Foundation, Melanoma Research Foundation, MacMillan Cancer Support, Cancer Compass, and Cancer Survivors Network.

### Data Preprocessing and Subsetting

Posts in the public domain on the included forums were programmatically extracted using validated algorithms in the R Programming Language. Upon extraction, data were deidentified by removal of identifiable personal information (ie, name, post or zip code, place names, email addresses, phone numbers, social security numbers, and conversion of raw usernames to unique identifiers). Data were also processed to correct for misspellings, remove non–Unicode Transformation Format-8 text, remove duplicate posts, and standardize all drug names to generic names.

Data were restricted to posts of users who began posting on or after the start of the study period and who mentioned at least one of the following treatments in their posts: binimetinib, dabrafenib, encorafenib, ipilimumab, nivolumab, pembrolizumab, or trametinib. Machine learning (ML) methods were used to predict whether posts contained actual treatment experiences as opposed to noise. Supervised ML algorithms were trained and tested on a random sample of over 1000 sentences from the collected data, which were manually labelled as “treatment experience related” or “not treatment experience related” to distinguish posts of interest and those containing noise. The best performing model was selected and applied to the data for subsequent analyses so that only users whose posts were predicted to contain actual treatment experiences were retained.

Natural language processing (NLP) methods (eg, inspection of clusters and n-grams) were used to stratify users into mutually exclusive adjuvant or metastatic groups based on lexical terms within posts. Terms derived from users’ posts were combined with those determined a priori (ie, “I had surgery” or “received adjuvant”) to generate the final list of terms for the population identification. The adjuvant group contained users with a mention of having surgery and no indication of metastatic disease, and the metastatic group consisted of users with terms relating to metastatic disease or treatments indicated at the metastatic setting. Users who could not be assigned to one of the groups were excluded from analyses. NLP methods using mentions of treatments in posts were used to further classify users into one of the following treatment subgroups:

- TT: dabrafenib/trametinib, encorafenib/binimetinib (metastatic group only)
- Immunotherapy: Pembrolizumab, ipilimumab/nivolumab (metastatic group only), or nivolumab

Treatment subgroups were not mutually exclusive, and posts were restricted to those containing the respective treatment to ensure the specificity of the data analyzed.

### Data Analysis

#### Symptom Identification

Symptoms were captured using the Apache Clinical Text Analysis Knowledge Extraction System (cTAKES) [19], a NLP tool that maps concepts from the Unified Medical Language System to clinical terms mentioned within posts. cTAKES was supplemented with custom lexicons to capture lay terms used by patients and caregivers (ie, nonclinical events). The custom lexicons were initially compiled by using the FDA Adverse Event Reporting System reports and further expanded upon inspection of the most frequently occurring lay terms used by users.

The output was manually inspected, and revisions were made where necessary to remove clinical terms incorrectly captured as symptoms a patient experienced. Rates of symptom occurrence were calculated as users with a co-occurrence of a symptom mention and treatment in the same post over the number of users with a mention of the treatment.

#### Qualitative Data Analyses

Manual qualitative data analysis (QDA) was performed to capture the impacts of symptoms and treatment discussed in the forum. Due to the large volume of posts, random samples of users were generated from the overall population included. Full posting histories from those users were qualitatively reviewed. This exercise was conducted separately for each treatment group. A random sampling strategy was used to include a holistic view of the experience of forum users. Qualitative coding was conducted in ATLAS.ti (version 8.4.4) by two researchers following thematic analysis principles, and codes were assigned to data-driven themes, categories, and subcategories [20,21]. The posts were coded until saturation was reached. Saturation was defined as the point at which no new categories of codes were generated by reviewing additional data. Codes and themes were reviewed by a researcher who did not code the data.

### Ethical Conduct

At the time of conducting the study, no strict guidelines on the appropriate use of health-related social media data had been developed. However, this study followed the recently published ethics framework from the University of Sheffield [22]. Only public open-access forums were used, where contents were openly visible and there was no requirement to register or to create a profile to view content. Terms and conditions of included forums were carefully reviewed to ensure compliance. To protect user privacy, no quotations are provided verbatim, and the original post cannot be traced in search engines using the text presented.

## Results

### Study Population

A total of 1037 users (9023 posts) and 114 users (442 posts) were included in the metastatic group and adjuvant group, respectively. A breakdown by treatment subgroup for each group is provided in Tables 1 and 2. As expected, given the timeline of treatment approvals, the largest treatment subgroups were nivolumab and pembrolizumab, and the smallest was encorafenib/binimetinib.

**Table 1:** Users included in the metastatic group by treatment group and analyses.

Treatment subgroup^a^	Symptom identification			Qualitative data analysis	
	Users, n	Posts, n	Users, n		Posts, n
Encorafenib/binimetinib	20	36	34		98
Dabrafenib/trametinib	215	659	18		30
Ipilimumab/nivolumab	499	2723	34		92
Nivolumab	443	3751	27		109
Pembrolizumab	451	3171	28		78

^a^The treatment groups are not mutually exclusive.

**Table 2:** Users included in the adjuvant group by treatment group and analyses.

Treatment subgroup^a^	Symptom identification		Qualitative data analysis	
	Users, n	Posts, n	Users, n	Posts, n
Dabrafenib/trametinib	18	41	10	27
Nivolumab	63	263	20	61
Pembrolizumab	45	209	24	100

^a^The treatment groups are not mutually exclusive.

### Identified Symptoms

**Figure 1:**
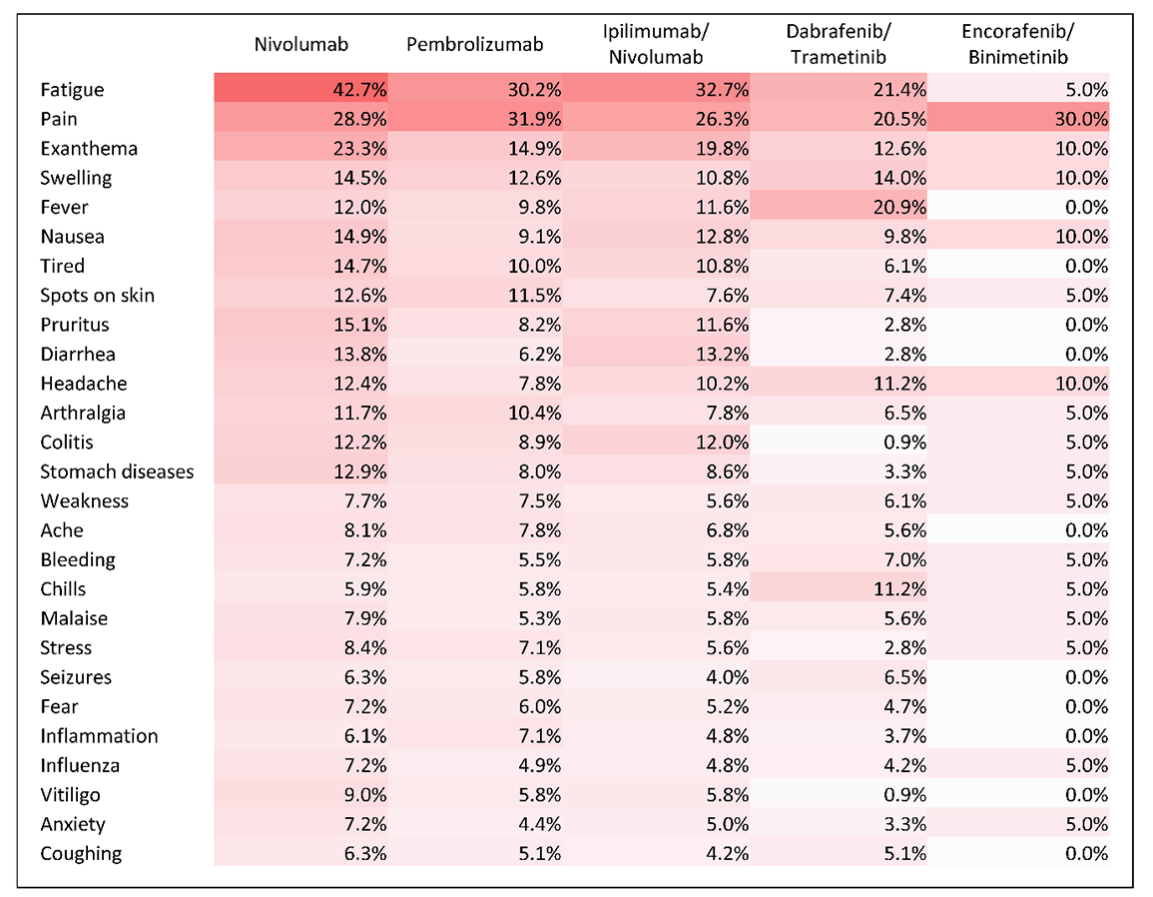
Heat map of the most mentioned symptoms, metastatic group, by treatment group.

**Figure 2:**
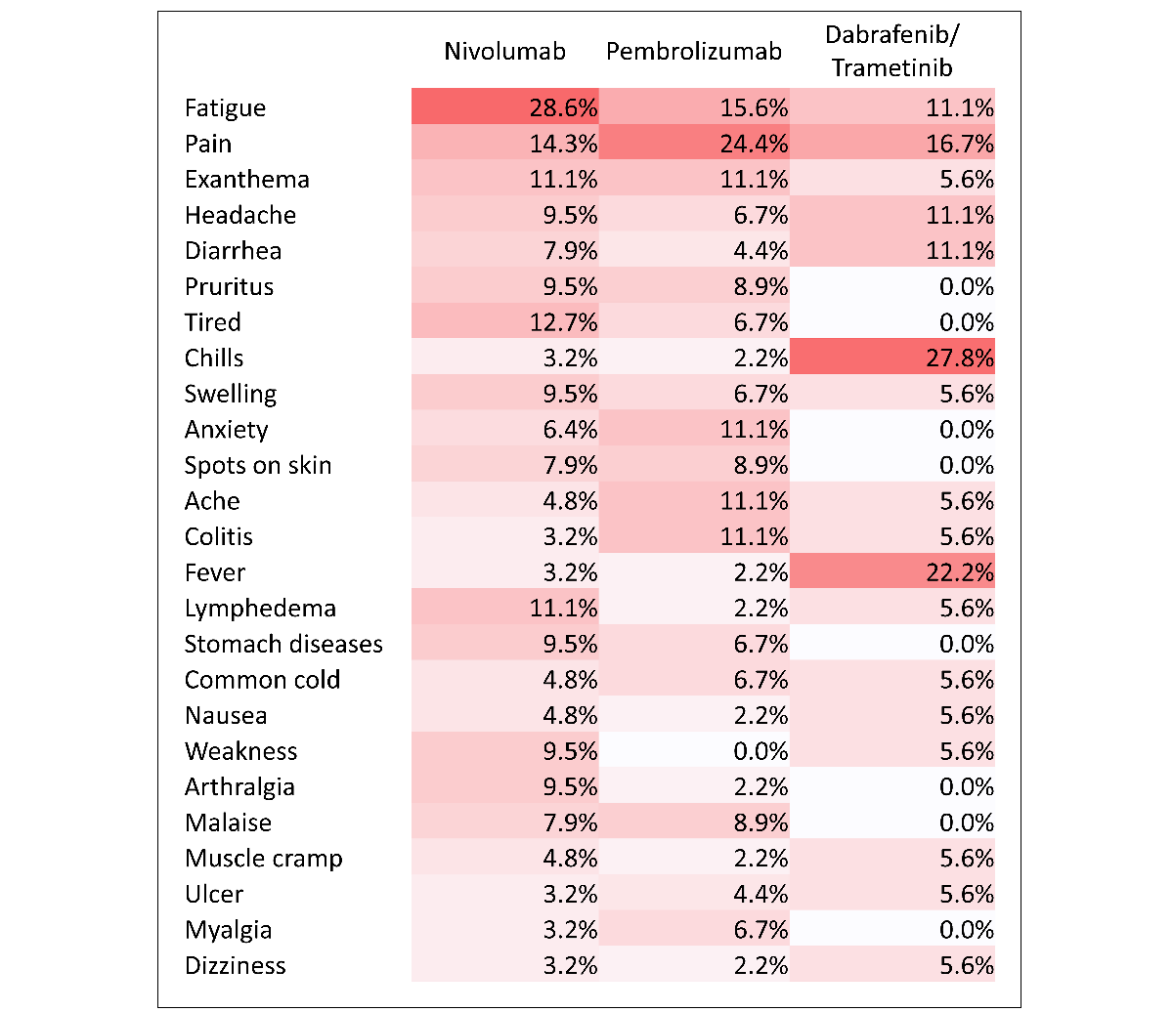
Heat map of the most mentioned symptoms, adjuvant group, by treatment group.

In both groups, fatigue, pain, or exanthema were the most mentioned symptoms by patients with metastatic melanoma or their caregivers in the forums (Figures 1 and 2).

In the metastatic group, fatigue was the most mentioned symptom for patients taking nivolumab (189/443, 42.7%), ipilimumab/nivolumab (163/499, 32.7%), and dabrafenib/trametinib (46/215, 21.4%), and pain was the most common symptom in pembrolizumab (144/451, 31.9%) and encorafenib/binimetinib (6/20, 30%). In the adjuvant group, fatigue and pain were the most common symptoms experienced by users in the nivolumab (n=18, 29%, and n=9, 14%, of 63 users, respectively) and pembrolizumab (n=7, 16%, and n=11, 24%, of 45 users, respectively) treatment groups, and chills and fever were the most common symptoms experienced in the dabrafenib/trametinib (n=5, 28%, and 4, 22%, of 18 users, respectively) treatment group.

### Symptom Impacts

#### Metastatic Group

Symptom impacts varied by treatment subgroup; however, physical and psychological effects were the most common negative impacts reported. Physical impacts included mobility issues, being unable to drive, and overall reduced activity. Psychological impacts included feelings of anxiety, depression, frustration, worry, and loss of dignity. Negative impact on social life was reported among the dabrafenib/trametinib and nivolumab treatment groups, and disturbed sleep was reported among the binimetinib/encorafenib, nivolumab, and ipilimumab/nivolumab treatment groups; however, they were reported less frequently. Patients in the immunotherapy and TT groups reported impacts on their physical ability, including their day-to-day tasks and ability to perform activities requiring mobility:

His quality of life has extremely deteriorated and he is now unable to perform physical activities

Caregiver

As treatment continues, I have developed panic attacks

Patient

#### Adjuvant Group

Findings for the adjuvant group were limited due to the small sample size; however, adverse impacts on family life (“She is too weak to enjoy spending time with her” [caregiver]) and physical impacts (“heel pain wasn’t bad at the start, but now I sometimes feel I can barely walk” [patient]) were identified. In addition, reduction of perceived quality of life (“the fatigue is really bothering the husband” [caregiver]) and impact on work (“I changed my work schedule as I was worried about side effects” [patient]) was reported [Table 3].

**Table 3:** Impacts of symptoms by group.

	Metastatic group	Adjuvant group
Physical impacts	Bedridden Less active Difficulty doing physical activity Difficulty exercising Difficulty getting out of bed Difficulty moving Difficulty walking General impact on quality of life Unable to drive	Inability to walk Taking a break from running Unable to exercise as before
Psychological impacts	Anxiety Concern Conflicted Depression Frustration Loss of dignity Nervous Panic attack Worried	Annoyance Frustration
Impacts on sleep	Difficulty sleeping Inability to stay awake for long	NR^a^
Impacts on social life	Needing to plan social outings Stopped socializing	NR
Family/caregiver burden	Feeling angry with patient Emotional impact to family	Inability to enjoy time with grandchildren
Impacts on work	Interruption to work	Changing work schedule Taking time off from work

^a^NR: not reported.

### Symptom Duration

#### Metastatic Group

Symptoms lasting less than a week appeared most common among patients receiving dabrafenib/trametinib, and longer-term sequelae appeared most common among patients receiving ipilimumab/nivolumab. Short-term symptoms (ie, those lasting up to 1 week) included fever, headache, fatigue, and soreness. Longer-term ones (ie, lasting longer than 1 week) included liver issues, nausea, diarrhea, and fatigue.

#### Adjuvant Group

More than one-third of patients receiving dabrafenib/trametinib or nivolumab mentioned symptom duration. Similar to the metastatic group, short-term symptoms appeared more frequently in patients receiving dabrafenib and trametinib, while longer-term issues were most commonly mentioned by patients receiving nivolumab. Examples of longer-term symptoms included liver problems, headache, colitis, and joint issues.

### Impacts of Treatment

#### Metastatic Group

In the metastatic group, the positive impacts mentioned by forum users included effects on their general health and quality of life, physicality, work, and social life or family. Patients mentioned feeling better and happier, and being able to continue life as normal because of their treatment. Positive physical effects included gaining weight, looking better, being able to exercise, and feeling stronger. Examples of the positive influences for TTs include:

I can work and complete tasks as usual

patient

This is the first time in months that I have felt like myself

patient

For immunotherapy:

As time progresses, he is getting stronger and gaining some weight. He is also doing some physical activity everyday

caregiver

I am able to spend time with friends and family as I now believe I have several more years to live

patient

Negative effects of treatments on social/family aspects and work included not being able to travel with family, partners wanting time off from job, and the patient having to work less.

#### Adjuvant Group

In the adjuvant group, forum users had discussions on improvements in general health and quality of life, work, and social/family. Patients reported they felt better, worked as usual, and spent more time with family. A user treated with TT expressed their personal family experience:

my grandson will be born soon. This treatment has made it possible for me to appreciate moments that I didn’t think I would see

**Figure 3:**
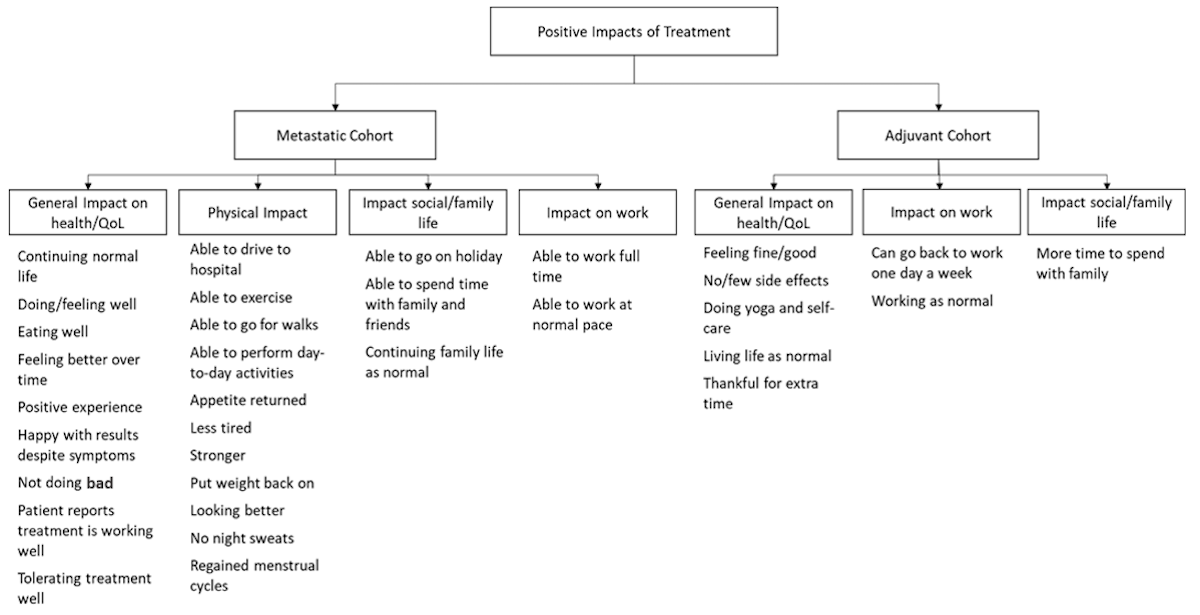
Positive impacts of treatment. QoL: quality of life.

In addition, a user who underwent immunotherapy reported the positive impact going to work had on their well-being: “I was still able to work, which made things seem normal.” Figure 3 illustrates the positive impacts of treatment in both groups.

## Discussion

### Principal Findings

This exploratory work uncovered the most common concerns discussed on social media forums among patients with adjuvant and metastatic melanoma receiving immunotherapy or TTs and their caregivers. Symptoms and related physical impacts (eg, inability to perform usual activities) were the most frequently reported issues in both cohorts, while psychological impacts (eg, anxiety) were also discussed among metastatic patients. Where discussed, treatment preferences were primarily focused on reduced risk of adverse events.

To the best of our knowledge, no published research has investigated experiences of patients with adjuvant or metastatic melanoma with immunotherapy and TT using health-related social media data.

Among the metastatic group, the most common symptom reported was fatigue in all treatment groups except for pembrolizumab and encorafenib/binimetinib, where pain was most common. Treatment included perceived improvements in general health, quality of life, physicality, ability to work, and patient’s social and family life. In the adjuvant group, comments reflected enhancements of general health, quality of life, work, and social/family interactions.

This study provides a unique perspective on patients’ experiences receiving immunotherapy or TTs. It is crucial to consider patient perspectives to ensure that real-life experiences and expectations are understood, especially as new therapies for melanoma become available. Social media not only allows patients to share their story but also provides a platform for patients and caregivers to seek support from others with similar experiences. This creates a sense of community that allows users to share positive experiences and burdens. Symptom impacts varied by treatment subgroups; however, the negative discussions generally featured work, family, and the physical aspects. The patients’ and caregivers’ point of view is not often incorporated in research but does play a large role in patients’ and their families’ well-being.

### Comparison to Prior Literature and Interpretation

A study that examined the extent to which social media health data could provide insight for relative effectiveness assessment concluded that, within oncology, these real-world data sources can be used to assess adverse events and evaluate quality of life [18].

To the best of the authors’ knowledge, this is the first published study examining the impacts of immunotherapy and TT among patients with melanoma and their caregivers using social media forums. The most commonly reported symptoms by patients with metastatic melanoma self-reporting taking pembrolizumab were pain, fatigue, and exanthema, which aligns with some of the common side effects previously reported [23,24]. Fatigue and skin problems are some of the common side effects of Nivolumab, which aligns with the first and third most common symptoms identified in this study, respectively [25]. The same common side effects were identified with ipilimumab-nivolumab again mapping to those symptoms identified in this study [26]. Fatigue, nausea, diarrhea, vomiting, and abdominal pain have been previously identified as common side effects of binimetinib in combination with encorafenib [27,28]; however, our study identified pain as the most mentioned symptom by patients with metastatic melanoma who self-reported receiving this treatment.

### Strengths and Limitations

Patients’ and caregivers’ firsthand experiences are potentially likely to reflect the true opinions of the users as they are provided spontaneously. Comments are possibly less likely to be impacted by information bias than traditional interview studies with no research or medical professionals present. This exploratory analysis provides insights as to which topics were most frequently discussed by patients with adjuvant or metastatic melanoma using forums receiving immunotherapy or TT. These are likely to reflect those of most importance to patients. The use of QDA allowed for further insight into factors of importance to patients and their caregivers, including those that may not have been considered at study conception.

The study was not free of limitations. First, due to the nature of the data and to respect patient privacy, the researchers were restricted by the amount of detail provided by users. Although all relevant detail on patients’ and caregivers’ experiences were coded during the qualitative review of posts, researchers could not ask for clarification in instances where users did not provide sufficient information; therefore, some detail may have been missed for a small number of users. Second, as the study was primarily exploratory in nature, all potentially relevant data were included in the analysis resulting in varied sample sizes across treatment groups. Thus, results from the QDA should be interpreted with caution as no statistical tests were conducted to assess differences between treatment groups. Findings were limited in the adjuvant group due to the small sample size, which is likely a result of recent approvals for the treatments of interest at this setting at the time of conducting the study. If repeated for a longer time after approval, a larger sample size could be achieved. Third, patients posting on forums cannot be considered representative of the entire melanoma patient population. Due to a lack of consistent reporting of patient attributes (eg, clinical and demographical characteristics), representativeness is challenging to assess in social media forums; however, this study was exploratory in nature with no comparative analyses. To capture a broad patient population and mitigate bias from nonrepresentativeness, multiple social media forums were included with no geographical restrictions. Finally, biases in health-related social media studies are not well understood, for example, the extent of information bias present in users’ posts. However, no study can be considered free of bias and such bias is not a limitation unique to this exploratory study.

### Conclusions

This exploratory study uncovered the most discussed symptoms and their associated impacts among patients and caregivers using health-related social media forums. This suggests that these are the topics of utmost importance to patients and caregivers influencing their lives. Future research should aim to validate and investigate less frequently discussed topics and could include patient questionnaires, interviews, or focus groups. Such studies could be used to assess how important these topics are to patients and caregivers, and to validate the findings of this study.

## Abbreviations

cTAKES: Clinical Text Analysis Knowledge Extraction System
FDA: Food and Drug Administration
ML: machine learning
NLP: natural language processing
QDA: qualitative data analysis
TT: targeted therapy
